# Genotypic variability and risk factors of hepatitis B virus in Western Siberia

**DOI:** 10.1186/1742-4690-8-S1-A211

**Published:** 2011-06-06

**Authors:** Roman Bayandin, Galina Kochneva, Galina Sivolobova, Antonina Grazhdantseva, Sergei Netesov

**Affiliations:** 1 State Research Center of Virology and Biotechnology “Vector", Koltsovo, Novosibirsk oblast, 630559, Russia; 2 Novosibirsk State University, Novosibirsk, 630090, Russia

## 

In the study were included one group of acute hepatitis patients (1043 persons) of Barnaul's city hospital and four groups (500 persons in each group) of Novosibirsk’s oblast inhabitants: medical staff, patients drug abuse clinic, AIDS center’s patients, clinic patients applied for any disease. The frequency of HBsAg was: 3.6% in group of polyclinic patients, 5.4% of medical workers, 8.4% among drug abuse clinic’s patients, 35% of AIDS center’s patients and 36.5% of Barnaul’s patients. Genotypes distribution was as follows: Novosibirsk oblast - 168 isolates of genotype D, 3 isolates of genotype A and genotype 2 isolates – C. In Barnaul: genotype D - 72 isolates, genotype A - 1. In the groups of Novosibirsk oblast the highest risk to be infected revealed for drug users (OR = 6.75). The risk of seropositivity was increased among respondents who had more than 4 sexual partners during lifetime (OR = 2.23); who had sex with HBV patients (OR = 2.12); who spent at least one night at prison (OR=2.58). Risk factors also found for men (OR=1.85) and young people (OR=1.79) as those who are more inclined to risky behavior. The biggest risk factors in Barnaul were found among men who have sex with men (OR=8.79), person with >2 sexual partners in last 6 month especially in women (OR=5.15), those who >30 years (OR=4.51). High risk had person who had contacts with blood (OR=4.05). In Novosibirsk and Barnaul different medical operations such as surgery, blood transfusion, overall anesthesia weren't show itself as risk factors.

